# Percentage depth dose evaluation in heterogeneous media using thermoluminescent dosimetry

**DOI:** 10.1120/jacmp.v11i1.2947

**Published:** 2010-01-28

**Authors:** L.A.R. da Rosa, S.C. Cardoso, L.T. Campos, V.G.L. Alves, D.V.S. Batista, A. Facure

**Affiliations:** ^1^ Instituto de Radioproteção e Dosimetria IRD/CNEN Rio de Janeiro Brasil; ^2^ Instituto de Física Universidade Federal do Rio de Janeiro Rio de Janeiro Brasil; ^3^ Instituto Nacional de Câncer INCA Rio de Janeiro Brasil; ^4^ Comissão Nacional de Energia Nuclear Rio de Janeiro Brasil

**Keywords:** thermoluminescent detector, lung heterogeneity, small field size, 15 MV, treatment planning system

## Abstract

The purpose of this study is to investigate the influence of lung heterogeneity inside a soft tissue phantom on percentage depth dose (PDD). PDD curves were obtained experimentally using LiF:Mg,Ti (TLD‐100) thermoluminescent detectors and applying Eclipse treatment planning system algorithms Batho, modified Batho (M‐Batho or BMod), equivalent TAR (E‐TAR or EQTAR), and anisotropic analytical algorithm (AAA) for a 15 MV photon beam and field sizes of 1×1,2×2,5×5, and $10\times 10\;{\text{cm}}^{2}$. Monte Carlo simulations were performed using the DOSRZnrc user code of EGSnrc. The experimental results agree with Monte Carlo simulations for all irradiation field sizes. Comparisons with Monte Carlo calculations show that the AAA algorithm provides the best simulations of PDD curves for all field sizes investigated. However, even this algorithm cannot accurately predict PDD values in the lung for field sizes of 1×1 and $2\times 2\;{\text{cm}}^{2}$. An overdosage in the lung of about 40% and 20% is calculated by the AAA algorithm close to the interface soft tissue/lung for 1×1 and $2\times 2\;{\text{cm}}^{2}$ field sizes, respectively. It was demonstrated that differences of 100% between Monte Carlo results and the algorithms Batho, modified Batho, and equivalent TAR responses may exist inside the lung region for the $1\times 1\;{\text{cm}}^{2}$ field.

PACS number: 87.55.kd

## I. INTRODUCTION

The use of small area photon radiation fields, mainly in the presence of low‐density heterogeneous materials, may become a complex scenario to be analyzed by the treatment planning system (TPS) in order to determine dose distributions. This is especially so if their heterogeneity correction algorithms are not able to consider the electronic lateral disequilibrium that takes place in such conditions.^(^ ^1^ ^,^ ^2^ ^)^ Narrow beams are presently very common in radiotherapy, mainly when new technologies are being employed as, for instance, intensity‐modulated radiation therapy (IMRT) and radiosurgery.^(^ ^3^ ^,^ ^4^ ^)^

Lung tissue is one of the most important low‐density heterogeneities that impact upon the success of a radiation therapy. That heterogeneity deserves special consideration not only because the absence of electronic equilibrium may interfere in the quality of the treatment planning (when narrow beams are involved in the process), but also due to the breathing motion of the lungs that is a factor of complexity during the therapeutic dose administration.^(5)^

Dose evaluations in the presence of narrow radiation beams are not easy. The small radiation field areas and the steep fluence gradients of beams require a detector with good spatial resolution. Several studies have been conducted concerning the dosimetry of narrow photon beams in a homogeneous medium.^(^ ^6^ ^–^ ^11^ ^)^ Different authors investigated the influence in dose distributions of lung‐equivalent materials exposed to such small area beams.^(^ ^1^ ^,^ ^2^ ^,^ ^12^ ^–^ ^14^ ^)^

Jones et al.^(13)^ studied the effect of lung heterogeneity on small beamlets for 6, 15 and 24 MV photon beams using the EGSnrc Monte Carlo code. Jones and Das^(14)^ studied depth dose data from the Monte Carlo simulations and compared them to the results provided by heterogeneity algorithms for a 6 MV beam. Three studies^(^ ^1^ ^,^ ^2^ ^,^ ^12^ ^)^ compared dosimetric measurements, Monte Carlo simulations, and treatment planning system calculations. Arnfield et al.^(12)^ studied the effects of lung heterogeneities on depth dose and lateral beam profiles for 6 and 18 MV photon beams. Field sizes of 4×4 and $10\times 10\;{\text{cm}}^{2}$ were investigated. Duch et al.^(2)^, by means of thermoluminescent (TL) dosimetry and Monte Carlo PENELOPE code, studied the reliability of Eclipse algorithms. They investigated a field size of $2\times 2\;{\text{cm}}^{2}$ and 6 and 10 MV photon beams. Carrasco et al.^(1)^ compared dose measurements, Monte Carlo simulations, and treatment planning system calculations for 10×10,5×5,2×2, and $1\times 1\;{\text{cm}}^{2}$ field sizes and X‐ray spectra of 6 and 10 MV. For the $1\times 1\;{\text{cm}}^{2}$ irradiation field, they did not obtain PDD curves using TL dosimetry.

The objective of this study was to compare percent depth dose (PDD) curves calculated using the radiation therapy Eclipse planning system version 8.1, applying its heterogeneity correction algorithms modified Batho (M‐Batho), general Batho (BPL), equivalent tissue‐air‐ratio (E‐TAR), and anisotropic analytical algorithm (AAA). These curves were measured using thermoluminescent dosimetry and simulated by Monte Carlo calculations.

The investigation was conducted for the 15 MV photon beam generated by a Clinac 2300 C/D and for irradiation field areas of 10×10,5×5,2×2, and $1\times 1\;{\text{cm}}^{2}$, using a soft tissue phantom with lung‐tissue equivalent material heterogeneity. The 15 MV photon beam was chosen because that is the quality used by the Brazilian National Institute of Cancer (where the present work was developed) for conventional lung cancer radiation therapy. Additionally, higher energy beams favor the investigation of the occurring physical processes; though a 6 MV photon beam could be more adequate for a radiation treatment involving narrow beams like, for instance, IMRT. The present work does not intend to generate correction factors for lung cancer treatments planned with the use of the Eclipse treatment planning system. Its objectives are to analyze the physical processes involved in the situation that include narrow beams and lung heterogeneity, and to evaluate the performance of the considered system under unfavorable complex situations.

## II. MATERIALS AND METHODS

PDD values at the presence of lung heterogeneity for irradiation fields of 10×10,5×5,2×2, and $1\times 1\;{\text{cm}}^{2}$ were experimentally determined employing thermoluminescent dosimeters and calculated using the Eclipse treatment planning system. The information obtained through TL dosimetry, when compared to planning system calculation, allowed the evaluation of the performance of the different Eclipse heterogeneity correction algorithms in calculating the patient treatment dose and, consequently, allowed the evaluation of the accuracy in delivering the therapeutic dose at the presence of heterogeneities.

The treatment planning system used was Eclipse. It calculates the dose with the pencil‐beam model or a heterogeneity correction factor by means of four algorithms: general Batho, modified Batho, equivalent TAR, and anisotropic analytical algorithm (AAA).^(^ ^15^ ^–^ ^20^ ^)^ All these algorithms were studied for the thorax phantom, considered in this work to be similar to lung heterogeneity.

This experiment had the objective of PDD determination considering the four irradiation field sizes considered in this work. The measurements were carried out using LiF:Mg,Ti thermoluminescent dosimeters and a geometric phantom specially projected for this purpose.

A thoracic geometric phantom was constructed using acrylic plates, simulating the soft tissue, and cork plates to simulate the lung tissue. Its total dimensions are similar to those of an adult human thorax. The phantom had a simple cubic regular form in order to facilitate the understanding of the physical processes of radiation interaction with matter.

Twenty‐one acrylic plates were used, of which 13 plates had dimensions of $30\times 30\times 1\;{\text{cm}}^{3}$, five plates had dimensions of $30\times 30\times 0.2\;{\text{cm}}^{3}$, and two plates had dimensions of $30\times 30\times 0.5\;{\text{cm}}^{3}$. Additionally, a forth plate type with dimensions of $30\times 30\times 0.2\;{\text{cm}}^{3}$, with two central holes, was used to place two TL detectors at different positions on the central axis of the radiation beam. The hole dimensions were similar to the TL detector dimensions in order to reduce as much as possible the existence of air gaps between the detector edge and the plate material. For Monte Carlo simulations, the possible presence of air gaps was disregarded. The complete acrylic system height is 15 cm.

Thirty‐six $30\times 30\;{\text{cm}}^{2}$ cork plates were employed in the montage of the lung phantom. Twenty‐two plates had a thickness of 0.4 cm; 14 plates were 0.3 cm thick. Additionally, there was a cork plate of $30\times 30\times 0.4\;{\text{cm}}^{3}$, with two central holes, which was used to place the TL detectors during the measurements. The hole dimensions were similar to the TL detector dimensions (for the same reasons as previously explained). The total phantom thickness agrees with the mean thickness of an adult man's lung.

**Figure 1:**
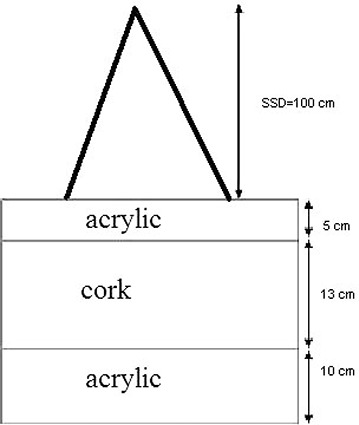
Schematic figure of the soft tissue/lung tissue phantom.

The plates were superposed so as to form an acrylic block of 5 cm high on a cork block of 13 cm high on an acrylic block of 10 cm high. Figure 1 shows a scheme of the phantom.

The irradiations were performed employing a Varian Clinac 2300 C/D linear accelerator. Photon beams with maximum energy of 15 MeV were used.

The Clinac gantry was always maintained at 0°. The dose rate used was 400 MU/minute. The monitor units were determined in order to obtain a dose of 1Gy at a depth of 3.0 cm, the depth of maximum dose for the photon spectrum considered in this work. The PDD curves were determined for all irradiation fields considered, namely 10×10,5×5,2×2, and $1\times 1\;{\text{cm}}^{2}$. A therapeutic ${}^{60}\text{Co}$ unit Theratron TH780 C was also used for TL dosimeter characterization, namely the dosimeter batch mean zero dose reading, the dosimeter batch lower detection limit, and the dosimeters individual sensitivity factors determination.

The thermoluminescent dosimeters used were lithium fluoride doped with magnesium and titanium (LiF:Mg,Ti) TLD‐100 (Saint‐Gobain Crystals & Detectors, Newbury, OH). They were chips with dimensions of $3.0\times 3.0\times 0.9\;{\text{mm}}^{3}$. Their evaluation was carried out in a PCL3 TL reader manufactured by the FIMEL Company (Fontenay Aux Roses, France). For annealing procedures, a TLDO automatic oven manufactured by PTW was used. As a reference dosimeter for TL dosimeter calibration, an IC70 clinic dosimeter and a 35040 Keithley electrometer were used.

**Figure 2:**
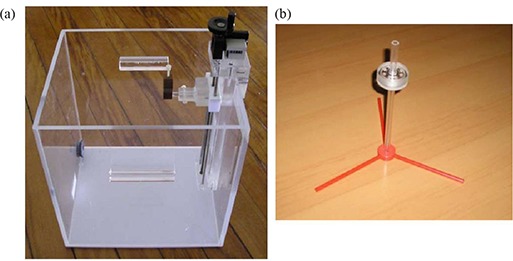
Geometric phantom (a) with dimensions of $38\times 38\times 30.5\;{\text{cm}}^{3}$, and (b) TL detector holder used for TL detector calibration.

For TL dosimeter calibration, different phantoms and holders were employed, namely two solid water plates of 5 cm thickness, two acrylic plates of $10\times 10\times 1\;{\text{cm}}^{3}$, a geometric acrylic phantom with dimensions of $38\times 38\times 30.5\;{\text{cm}}^{3}$ (CNMC, Nashville, TN), and an acrylic TL detector holder (Institute of Radiation Protection and Dosimetry, Brazil). In Figs. 2(a) and 2(b), the geometric phantom and the TL detector holder, respectively, are presented.

It was necessary to obtain the computerised tomography (CT) images of the thoracic phantom. For this purpose, a PQ 2000 CT scanner was employed. The images were used in the Eclipse treatment planning system.

The phantom was validated comparing the Hounsfield units for lung tissue versus cork and Hounsfield units for acrylic versus water. The tomography was executed in slices of 3 mm each, in a total range of 100 slices. The high voltage used was 130 kV.

The annealing procedure applied to the TL dosimeters was the very well‐established procedure consisting of a pre‐irradiation annealing of 400°C for 1 hour, followed by a heating procedure of 100°C for 2 hours, and a post‐irradiation annealing of 100°C for 15 minutes.^(^ ^21^ ^–^ ^24^ ^)^ These procedures was carried out in the automatic oven PTW TLDO.

Sixty TL dosimeters were used in this work, of which 52 samples were applied in measurements, while the eight remaining dosimeters were used in the stability control of the TL system.

The mean zero dose reading, ${\text{TL}}_{0}$, the lower detection limit, and the individual sensitivity factors, $S_{i}$, were determined for all dosimeters. As individual calibration factors were not used, the utilization of sensitivity factors was important in order to minimize the differences of TL dosimeters sensitivities. The calibration procedure adopted was a first‐degree calibration curve relating TL reading and dose.

Eight TL dosimeters were used in order to obtain stability control correction factors. They were always exposed to 1 Gy using the 15 MV photon field produced by the Varian Clinac 2300 C/D. A first, irradiation was carried out and considered as reference. A mean reference reading was determined, ${\text{TL}}_{\text{ref}}$, which is given by Eq. (1):

(1) $${\text{TL}}_{\text{ref}}=\frac{\sum_{i=1}^{8}\frac{\left({\text{TL}}_{i}-{\text{TL}}_{0}\right)}{S_{i}}}{8}$$

where ${\text{TL}}_{i}$ is the TL reading of the i‐th TL dosimeter, and $S_{i}$ is the sensitivity factor of the i‐th TL dosimeter.

Every time the measurement TL dosimeters were evaluated, control dosimeters exposed to 1 Gy were evaluated as well, in order to generate correction factors, ${\text{FC}}_{m}$, for the measurement dosimeters, according to Eq. (2):

(2) $${\text{FC}}_{m}=\frac{\sum_{i=1}^{8}\frac{({\text{TL}}_{im}-{\text{TL}}_{0})/S_{i}}{8}}{{\text{TL}}_{\text{ref}}}$$

where ${\text{TL}}_{\text{im}}$ is the TL reading of the i‐th TL dosimeter for the m‐th evaluation.

For the purpose of determining the calibration curve for the TL dosimeters, they were exposed in groups of eight dosimeters to 0.25, 0.5 and 1 Gy to water of 15 MV photon beam generated by the Varian Clinac 2300 C/D. The dosimeters were irradiated in the holder (as shown in Fig. 2(b)) placed inside the water‐filled acrylic geometric phantom (as shown in Fig. 2(a)). The TL readings were corrected by ${\text{TL}}_{0}$, $S_{i}$ and ${\text{FC}}_{m}$, according to Eq. (3):

(3) $${\text{TL}}_{\text{corr}(i\text{Dx})}=\frac{\left({\text{TL}}_{i\text{Dx}}-{\text{TL}}_{0}\right)}{S_{i}}\cdot {\left({\text{FC}}_{m}\right)}^{-1}$$

where ${\text{TL}}_{\text{corr}(\text{iDx})}$ is the corrected reading of the i‐th dosimeter for the dose $D_{x}$. Each experimental point of the calibration curve is the mean value of eight corrected TL readings. Experimental points were fit by linear function.

In order to determine PDD values, two TL dosimeters were used at each phantom depth and the TL reading considered for dose calculation, $\bar{{\text{TL}}_{\text{cor}}}$, was the average of the corrected individual readings of each TLD. The absorbed dose to water, $D_{w}$, for the depth considered is calculated through the calibration curve equation (Eq. (4)):

(4) $$D_{w}=A\bar{T}L_{\text{cor}}^{{\;}^{\bar{\;\;\;\;\;\;\;}}}+B$$

where A and B are, respectively, the angular and linear coefficients of the calibration curve. The uncertainty in absorbed dose‐to‐water assessment is determined by the propagation of individual uncertainties.

As a means to obtain an accurate experimental determination of PDD curves in the presence of the lung heterogeneity, two kinds of corrections to the experimental data were necessary, namely, a correction of the dose measurements in acrylic and cork, because the TL dosimeters were calibrated in terms of dose in water, and a correction of the dose measurements due to the presence of the TL dosimeter, which is itself a heterogeneity to the media. The dose to water was converted to dose to the considered medium, acrylic or cork, applying the Bragg‐Gray theory.^(25)^

In order to determine PDD values in a phantom containing a soft tissue equivalent material (acrylic) and a lung equivalent material (cork), it was necessary to convert dose‐to‐water values to dose‐to‐acrylic values and dose‐to‐lung tissue values, depending on the phantom region considered. This conversion was made on the basis of Bragg‐Gray theory, presented by Eq. (5):

(5) $$\frac{D_{w}}{D_{g}}=\frac{m\bar{S_{w}}}{m\bar{S_{g}}}$$

where $m\bar{S_{w}}$ is the mass collision stopping power for water and ${\text{mS}}_{g}$ is the mass collision stopping for the material of interest. $D_{w}$ and $D_{g}$ are the absorbed dose to water and to the material of interest (in the present case, acrylic or lung tissue, respectively). Mass collision stopping power values for cork were not found in the literature and the values considered in that case were those used for lung tissue. The energy considered for mass collision stopping power determination for acrylic and lung‐tissue (cork), $E_{0}$, was calculated using Eq. (6):^(26)^

(6) $$E_{0}=\frac{1}{2}h\upsilon \frac{{\;}_{e}\sigma_{\text{tr}}}{{\;}_{e}\sigma }$$

where hν is the mean energy of the photon spectrum, $e\sigma_{\text{tr}}$, is the differential cross section of energy transference per electron and eσ is the Klein‐Nishida cross section (Compton effect) per electron.

As the TL dosimeter behaves like heterogeneity for the medium where the absorbed dose should be evaluated, modifying the dose profile locally, it was necessary to evaluate this influence. Monte Carlo simulation was used with this purpose, and correction factors were generated and applied to the experimental results. The correction factors were obtained by employing the algorithm EGSnrc.^(^ ^27^ ^,^ ^28^ ^)^ A geometry of slices was simulated, including the own TL dosimeter as a heterogeneity through its chemical composition.

PDD curves were determined by Monte Carlo simulation in two situations, namely considering the TLD presence and not considering the TLD presence. For each point of experimental evaluation with TL dosimeter, a correction factor, $F_{\text{cor}}$, was calculated using the ratio of PDD values for the point of interest, given by Eq. (7):

(7) $$F_{\text{cor}}=\frac{{\text{PDD}}_{\text{TLD}}}{\text{PDD}}$$

where ${\text{PDD}}_{\text{TLD}}$ is the PDD value at the point of interest, determined by Monte Carlo simulation – considering the presence of the TLD and PDD is the PDD value determined by Monte‐Carlo simulation at the same point without considering the TLD presence.

Monte Carlo simulations of PDD at the presence of lung heterogeneity were performed using the DOSRZnrc^(27)^ user code of EGSnrc.^(28)^ The code DOSRZnrc is a general purpose Monte Carlo transport package, which can be used in a wide variety of applications besides radiotherapy beams. The code features include the ability to score the dose in cylindrical geometry. As in the treatment planning system, the doses were calculated for each square field used in this study. In order to apply the code DOSRZnrc that uses cylindrical geometries, square fields were converted into circular fields with the same area from a point source located 100 cm from the surface of the phantom. The conversion was carried out according to Eq. (8), ^(29)^ that preserves the scattering conditions of the square fields. The 15 MV incident X‐rays spectrum used for Monte Carlo simulations was determined by Mohan et al.^(30)^

(8) $$R=L.{\left(\phi \right)}^{1/2}$$

where L is the length of the edge of the square field and R is the radio of the equivalent circular field used in the simulations.

The global photon energy cutoff value was 0.1 keV and the cutoff energy for electrons was 70 keV. For simulations, a layered phantom consisting of acrylic and lung was created. The lung material used was LUNGICRU521, defined in Report 37 of the International Commission on Radiation Units and Measurements^(31)^ and the material used to simulate acrylic was PMMA from ICRU521.^(31)^

The dose was calculated for 60 slabs of 0.5 cm thickness along a cylinder located at the central axis of the beam with field size chosen to have the same area of a TLD chip $\left(3.0\times 3.0\times 0.9\;{\text{mm}}^{3}\right)$. The simulated depth dose curves were normalized at the depth of maximum dose.

The number of histories generated was sufficient to produce a statistical variance of less than 0.5% in the dose‐per‐incident fluence at the plane of maximum dose for each of the Monte Carlo simulations.

## III. RESULTS

Hounsfield unit values vary between −510 and −533HU for cork, and from −910 up to −500HU for lung tissue.^(32)^ These results indicate that cork can be used as an adequate material to simulate lung tissue.

Equation (9) was obtained for the TL dosimeter calibration curve:

(9) $$D_{w}=0.0009\;\;\bar{{\text{TL}}_{\text{cor}}}+1.4243$$

where $D_{w}$ is the absorbed dose in water in cGy and $\bar{{\text{TL}}_{\text{cor}}}$ is the average of the corrected individual readings of the two TL dosimeters, as defined previously.

The dose to water was converted to dose to the considered medium, acrylic or cork, applying the Bragg‐Gray theory. Stopping power ratios water‐acrylic and water‐lung tissue are, respectively, 0.966 and 0.988. These values are similar to those calculated by Siebers et al.^(33)^ using EGSnrc Monte Carlo code.

The total uncertainty associated to the experimental PDD determination was calculated through the propagation of specifics uncertainties as being less than 5%.

**Figure 3:**
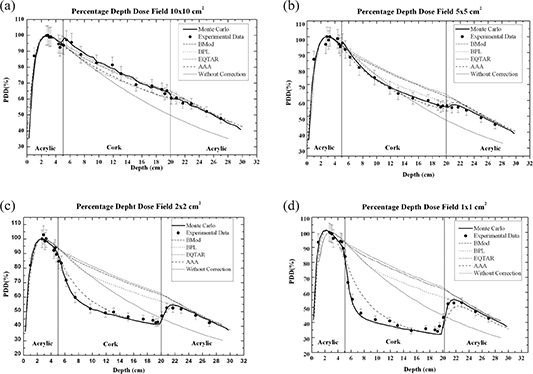
PDD curves calculated through the radiotherapy Eclipse planning system algorithms Batho, modified Batho, equivalent TAR, and anisotropic analytical algorithm (AAA); measured with thermoluminescent dosimetry and simulated using EGSnrc Monte Carlo code for a irradiation field of (a) $10\times 10\;{\text{cm}}^{2}$, (b) $5\times 5\;{\text{cm}}^{2}$, (c) $2\times 2\;{\text{cm}}^{2}$, and (d) $1\times 1\;{\text{cm}}^{2}$.

Figures 3(a), (b), (c) and (d) present the PDD curves simulated by Monte Carlo method, measured with thermoluminescent dosimetry and calculated through the radiotherapy Eclipse planning system version 8.1, respectively, for irradiations fields of 10×10,5×5,2×2, and $1\times 1\;{\text{cm}}^{2}$.

**Figure 4:**
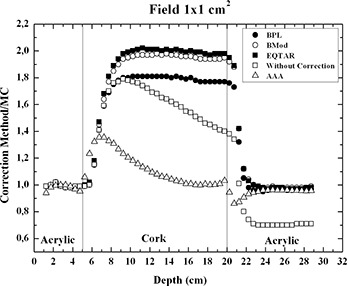
Ratio PDD results obtained with treatment planning system Eclipse version 8.1 to PDD results obtained with Monte Carlo, for $1\times 1\;{\text{cm}}^{2}$ field size.

**Figure 5:**
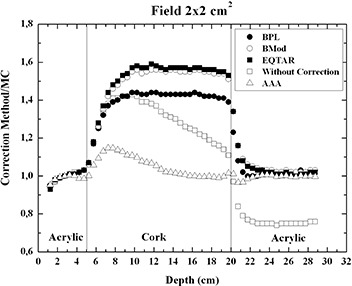
Ratio PDD results obtained with treatment planning system Eclipse version 8.1 to PDD results obtained with Monte Carlo, for $2\times 2\;{\text{cm}}^{2}$ field size.

It is interesting to observe that the experimental results agree with Monte Carlo simulations for all irradiation fields. In the cases of irradiation field areas of 1×1 and $2\times 2\;{\text{cm}}^{2}$, the experimental results of PDD, when compared to the values of PDD obtained with the planning system without correction, showed a better agreement than when compared to the corrected values of PDD obtained with the planning system based on the algorithms modified Batho, Batho, and E‐TAR. For the AAA algorithm, it could be observed that the PDD values are still overestimated in lung for a 15 MV beam, for the same small field sizes, although the best agreement is obtained. Figures 4 and 5 present the ratio PDD results obtained with treatment planning system Eclipse to PDD results obtained with Monte Carlo to 1×1 and $2\times 2\;{\text{cm}}^{2}$ fields sizes, respectively. The AAA algorithm cannot properly calculate the PDD value near the interfaces lung/soft tissue and, especially, soft tissue/lung. For the field $1\times 1\;{\text{cm}}^{2}$ at the interface soft tissue/lung, there is an over‐estimate of the dose in the lung of about 40%, and at the interface lung/soft tissue there is an under‐estimate of the dose in soft tissue around 18%.

## IV. DISCUSSION

The behavior presented by PDD curves obtained using thermoluminescent dosimetry with the narrowest beams (1×1 and $2\times 2\;{\text{cm}}^{2}$) can be explained based on the lateral electronic disequilibrium effect that is important for small fields, mainly in the low density medium, as presented in Figs. 3(d) and (c), respectively. When the distance from a point of interest within a field to the field edge is equal to or smaller than the Compton range for a given energy, the Compton interaction produces an electron that can transfer its energy to a point outside the radiation field. When the range of the Compton electrons generated is half the size of the irradiation field, any interaction will produce an electron that can transfer its energy to a point outside the radiation field. Therefore, even those interactions occurring on the central beam axis generate electrons that are not replaced by other electrons generated elsewhere in field. The electronic equilibrium is thus lost. Moreover, due to the wider range of these electrons in a low‐density material, compared to a water‐equivalent material, the lack of lateral electronic equilibrium occurs more predominantly in the form case, with a consequent reduction of the absorbed dose values inside the low density material. For the $5\times 5\;{\text{cm}}^{2}$ field, the electronic disequilibrium is greatly reduced, although its presence can be still detected (Fig. 3(b)). For the largest field, $10\times 10\;{\text{cm}}^{2}$, the effect is not present or is negligible.

The PDD curves determined by the Eclipse planning system, except for the AAA algorithm, differ from the experimental PDD curves because the heterogeneity correction algorithms do not take into account the transport of electrons and, therefore, are not able to evaluate the lateral electronic disequilibrium.

Comparisons with Monte Carlo calculations show that the AAA algorithm cannot accurately predict PDD values in lung for small field sizes like 1×1 and $2\times 2\;{\text{cm}}^{2}$, mainly at the interfaces soft tissue/lung and lung/soft tissue. Considering the smallest field, $1\times 1\;{\text{cm}}^{2}$, a 40% over‐dosage in lung is predicted by the planning system near the interface soft tissue/lung, and an 18% under‐dosage in soft tissue is presented by the planning system near the interface lung/soft tissue (Fig. 4). For the $2\times 2\;{\text{cm}}^{2}$ field, only a 20% over‐dosage in lung is predicted by the AAA algorithm near the interface soft tissue/lung. For other phantom regions, there is a good agreement between TLD results and AAA algorithm results even close the interface lung/soft tissue (Fig. 5).

In Fig. 3, it is possible to observe that differences of 100% between TLD results and treatment planning system results (except for the AAA algorithm) exist inside the low‐density region. It is important to mention that $1\times 1\;{\text{cm}}^{2}$ is as the smallest beam segment applied in intensity‐modulated radiotherapy. These differences may conduct to a considerable under‐dosage to the lung tissue. For the $10\times 10\;{\text{cm}}^{2}$ field, TLD results and Eclipse results present a good agreement.

## V. CONCLUSIONS

The results presented in this work indicate that close attention should be paid concerning the use of the Eclipse treatment planning system with the heterogeneity correction algorithms E‐TAR, Batho, M‐Batho, and AAA, for dose calculations involving small area irradiation fields and heterogeneities containing low‐density media. In the case of the narrowest beam contemplated in the present study $\left(1\times 1\;{\text{cm}}^{2}\right)$, the lowest deviations between TLD and planning system results are achievable if the AAA algorithm for heterogeneity correction is utilized.
